# Variation of Serum Lycopene in Response to 100% Watermelon Juice: An Exploratory Analysis of Genetic Variants in a Randomized Controlled Crossover Study

**DOI:** 10.1093/cdn/nzaa102

**Published:** 2020-06-17

**Authors:** Kristi M Crowe-White, Venkata S Voruganti, Valentina Talevi, Tanja Dudenbostel, Vinoth A Nagabooshanam, Julie L Locher, Amy C Ellis

**Affiliations:** Department of Human Nutrition, University of Alabama, Tuscaloosa, AL, USA; Department of Nutrition and Nutrition Research Institute, University of North Carolina-Chapel Hill, Kannapolis, NC, USA; Department of Nutrition and Nutrition Research Institute, University of North Carolina-Chapel Hill, Kannapolis, NC, USA; Cardiovascular Disease, Vascular Biology & Hypertension, University of Alabama at Birmingham, Birmingham, AL, USA; Nutrition Obesity Research Center, University of Alabama at Birmingham, Birmingham, AL, USA; Division of Gerontology, Geriatrics, and Palliative Care, University of Alabama at Birmingham, Birmingham, AL, USA; Department of Human Nutrition, University of Alabama, Tuscaloosa, AL, USA

**Keywords:** lycopene, watermelon, nutrigenomics, single nucleotide polymorphisms, carotenoids, cholesterol, antioxidant capacity, postmenopausal women

## Abstract

**Background:**

Watermelon, a rich source of lycopene, has garnered attention for cardioprotective effects including cholesterol reduction and promotion of redox balance. It is unknown whether 100% watermelon juice may represent a food-first approach to confer cardioprotective benefits of lycopene.

**Objectives:**

This study examined influences of 100% watermelon juice on serum lycopene, lipids, and antioxidant capacity. Secondly, the study explored genetic influences on lycopene metabolism and bioavailability.

**Methods:**

A placebo-controlled, randomized, double-blind, crossover trial with postmenopausal women (*n* = 16, mean ± SD age: 60 ± 4.1 y) assessed effects of 100% watermelon juice on mechanistic and clinical outcomes influencing vascular function. Participants maintained low-lycopene diets for a 1-wk run-in period and throughout the study. Morning and evening consumption of 100% watermelon juice provided a daily dose of 14.4 ± 0.34 mg lycopene. Study arms of 4 wk were separated by a 2-wk washout period. Saliva was collected for genetic analysis of single nucleotide polymorphisms, and fasting blood samples were taken pre– and post–study arms. Statistical analyses included mixed models, linear regression, and nonparametric tests.

**Results:**

Serum lycopene exhibited a significant treatment effect (*P* = 0.002) along with notable interindividual responses; however, significant improvements in serum lipids or antioxidant capacity were not observed. Genetic variant rs6564851 in the β-carotene 15,15’-oxygenase-1 (*BCO1*) gene was associated with changes in lycopene such that TT homozygotes exhibited a significantly greater increase (β ± SE: 13.4 ± 1.6, *P* = 1.4 × 10^−06^).

**Conclusions:**

Watermelon juice supplementation did not result in improvements in serum lipids or antioxidant capacity; however, results support findings in which watermelon juice significantly, yet differentially, increased circulating lycopene. Genetics appears to explain some of the variability. Given that dose has been shown to overcome individual responsiveness to lycopene interventions, future investigations with varying doses of lycopene-rich foods would be strengthened by genotyping so as to establish personalized nutrition recommendations.

This trial was registered at clinicaltrials.gov as NCT03626168.

## Introduction

Lycopene is among the most abundant carotenoids in human serum, and research suggests that it has superior singlet oxygen quenching potential compared with other carotenoids (1–4). The acyclic, highly conjugated hydrocarbon structure of lycopene imparts direct antioxidant functionality and indirect functionality through modulation of antioxidant enzyme production, thus limiting the oxidation of LDL cholesterol (5, 6). Lycopene also exerts other cardioprotective effects by inhibiting the rate-limiting enzyme in cholesterol synthesis and augmenting the activity of macrophage LDL cholesterol receptors similarly to the effect of statin drugs (7, 8). Nevertheless, mechanisms underpinning the effect of lycopene on vascular health may be attenuated by numerous factors known to influence lycopene bioavailability.

Among these factors, food processing, diet composition, adiposity, BMI, gender, and smoking account for ∼25% of the variance in lycopene bioavailability (9–13). Additional factors influencing the bioavailability and efficacy of lycopene include the form of consumption: food compared with supplement. For example, a recent meta-analysis of 21 intervention trials reported significant reductions in LDL cholesterol and improvements in flow-mediated dilation among interventions supplementing tomato products into the diet (14); however, systolic blood pressure was the only cardiovascular disease risk factor significantly reduced when lycopene supplements served as the intervention vehicle. Lastly, albeit understudied, studies suggest several genetic variants involved in carotenoid metabolism may influence lycopene bioavailability (**Table 1**) (12, 15–21).

**FIGURE 1:**
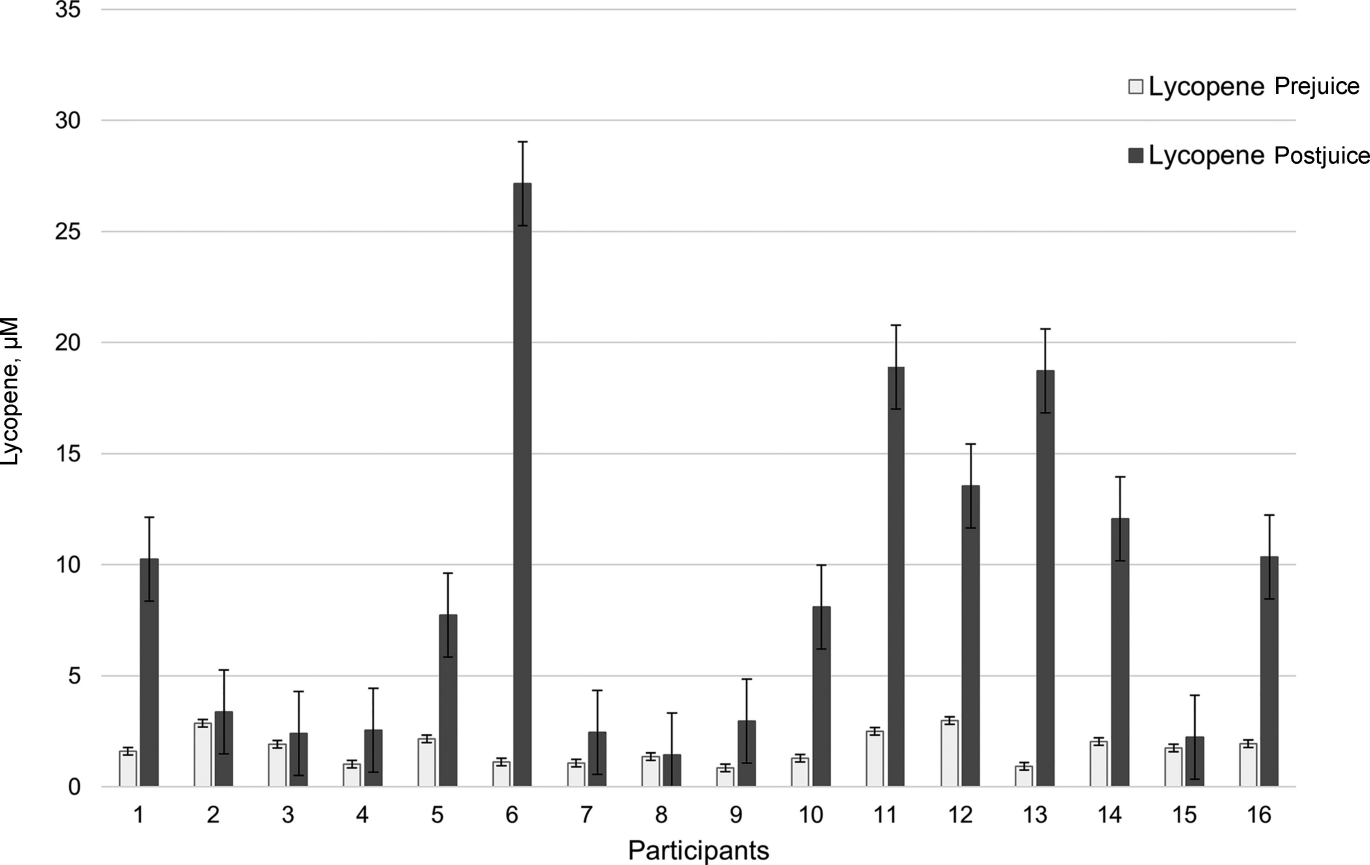
Interindividual differences in serum lycopene in response to 100% watermelon juice supplementation.

**TABLE 1:** Selected SNPs related to carotenoid bioavailability^1^

SNP	Gene	Allele	MAF	Supporting literature
rs7841189	*LPL*	C>T	14.8%	(15)
rs9468304	*ELOVL2*	G>A	30.2%	(15)
rs911196	*ELOVL2*	T>G	25.2%	(15)
rs9365046	*SOD2*	G>A	16.9%	(15)
rs6564851	*BCO1*	T>G	47.6%	(17, 19)
rs4112274	*CD36*	C>T	22.4%	(15)
rs1871744	*ABCG2*	T>C	17.3%	(15)
rs17482753	*LPL*	G>T	8.8%	(15)
rs17029173	*MTTP*	T>G	13.5%	(15)
rs1672879	*SCARB1*	G>A,T	26.9%	(12)
rs11057841	*SCARB1*	C>T	17.9%	(20)
rs1042031	*APOB*	C>A,T	12.8%	(15)
rs10248420	*ABCB1*	A>G,T	34.7%	(15)

1 SNP = single nucleotide polymorphism; *LPL*= lipoprotein lipase; *ELOVL2*= ELOVL fatty acid elongase 2; *SOD2*= superoxide dismutase 2; *BCO1*= beta-carotene oxygenase 1; *CD36*= CD36 molecule; *ABCG2*= ATP binding cassette subfamily G member 2; *MTTP*= microsomal triglyceride transfer protein; *SCARB1*= scavenger receptor class B member 1; *APOB*= apolipoproteinB; *ABCB1*= ATP binding cassette subfamily B member 1; MAF = minor allele frequency from the default global population of the 1000Genome Project (21).

Watermelon, a rich source of dietary lycopene, has garnered attention for its potential cardioprotective effects (5). In addition, intervention studies with other lycopene-rich foods have reported reductions in cholesterol and lipid peroxidation—outcomes associated with cardiovascular disease (22). Taken collectively, these findings mean watermelon may represent a food-first approach to confer cardioprotective benefits of lycopene. Thus, the objective of this study was to assess the influence of 100% watermelon juice supplementation on serum lycopene, lipids, and antioxidant capacity. Secondarily, the study aimed to explore potential genetic influences on lycopene metabolism and bioavailability.

## Methods

A placebo-controlled, randomized, double-blind, crossover trial (NCT03626168) with postmenopausal women (*n* = 16, mean ± SD age: 60 ± 4.1 y) assessed the effect of 100% watermelon juice on mechanistic and clinical outcomes influencing vascular function (23). By study design, participants were normotensive, nonsmokers with a BMI of 18.5–29.9 kg/m^2^, and in overall good health. Exclusion criteria included food allergy to watermelon; history of hypotension or hypertension; chronic kidney disease; diabetes; previous cardiac events and procedures; smoking or other tobacco use within the past 6 mo; use of anticoagulant, cholesterol-lowering, or blood-pressure medications; and use of dietary supplements containing antioxidants or bioactive compounds within watermelon. All protocols were approved by the Institutional Review Board at the University of Alabama, and all participants provided written informed consent for voluntary involvement in the study. Compensation was provided to participants at the beginning and end of each 4-wk study arm.

For a 1-wk run-in period and during the study, participants were asked to adhere to their typical diet pattern apart from foods rich in lycopene. Participants were provided with a list of lycopene-rich foods and were asked to limit these to 2 servings/d for the study duration including during the 2-wk washout period. Compliance to the low-lycopene diet was assessed by 3 random 24-h recalls that were analyzed using the Nutrition Data System for Research (Nutrition Coordinating Center, Minneapolis, MN, 2015), a software program designed for analysis of food diaries and 24-h dietary recalls. Diet recalls were administered by trained study staff using a multipass method validated for use in older adults (24). The 4-wk intervention arm consisted of morning and evening consumption of pasteurized 100% watermelon juice with a meal containing dietary lipids. The daily dose (2 bottles each containing 360 mL) provided 14.4 ± 0.34 mg lycopene. Selection of pasteurized 100% watermelon juice as opposed to watermelon fruit was based on the fact that thermal processing converts lycopene to its most bioavailable form: *cis*-lycopene (25). Pasteurized watermelon juice was donated by Frey Farms from a single harvest of Estrella variety melons. Samples from each batch of watermelon juice were tested for lycopene concentration to ensure homogeneity between batches. The 4-wk placebo arm consisted of morning and evening consumption of a placebo beverage formulated with sucrose, nonnutritive watermelon flavoring, pectin, cellulose, malic acid, and FDA-approved food coloring. The sugar content and acidity of the placebo beverage were matched to the 100% watermelon juice. The placebo beverage was devoid of bioactive compounds found in 100% watermelon juice.

To ensure participant blinding to treatment group, a sensory study was conducted with postmenopausal women to comparatively assess the sensory attributes of 100% watermelon juice and the watermelon-flavored placebo beverage (26). Blinding for the sensory study was achieved by randomly generated numeric sample codes along with randomized sample introduction using SIMS Sensory Quality Panel Software Systems. The lack of significant differences noted between the 100% watermelon juice and placebo in the sensory attributes of aroma, taste, and texture strengthens the double-blind crossover design of the present study by reducing participant discrimination and, possibly, improving adherence to the study protocol.

Participants were randomly assigned to the order in which they received juice or placebo using a randomization plan generated by PROC PLAN in SAS version 9.4 (SAS Institute), and a closed envelope method was used to assign a randomization code to each participant. Only research staff not involved in data collection or analysis had access to the randomization scheme, and those individuals were responsible for providing participants with their respective beverages. Before each 4-wk study arm, participants were provided with juice or placebo in 360 ml opaque bottles to be stored under refrigeration until time of consumption. Beverage compliance was assessed by two 28-d participant-administered log forms with check-off boxes for each dose, and minimal adherence was defined as 70% a priori. Saliva samples were collected for single nucleotide polymorphism (SNP) genotyping, and fasting blood samples were collected pre– and post–intervention arms.

### Assessment of serum lycopene, lipids, oxidized LDL cholesterol, and antioxidant capacity

Serum was stored at −80°C until analysis, at which time it was thawed and handled under dim light. Lycopene extraction was conducted according to a previously validated method (27). Briefly, 0.5 mL ethanol containing 1 g butylated hydroxytoluene/L was added to 0.5 mL serum and mixed on a vortex. Serum samples were extracted twice with 1.0 mL hexane. The organic layer was pooled and evaporated at 38°C under nitrogen for reconstitution with 2-propanol. The chromatographic separation was carried out using an ACQUITY ultra-HPLC system with a photodiode array detector and ACQUITY BEH Shield RP18 column (2.1 × 100 mm, 1.7 µm) (Waters) according to a previously described method for fat-soluble micronutrients (28). This method was selected owing to its excellent chromatographic reproducibility and sensitivity. Lycopene and β-apo-8’-carotenal were sourced from Sigma-Aldrich, and β-apo-8’-carotenal served as the internal standard for measurement. The limit of measurement for lycopene was 0.039 µM. Samples were extracted in duplicate and all samples were run in random order.

Blood lipids were measured on a Stanbio Sirus automated analyzer. Total cholesterol, triglycerides, LDL cholesterol, and HDL cholesterol levels are reported as milligrams per deciliter. Oxidized LDL cholesterol was analyzed using an enzyme immunoassay (Cell BioLabs, Inc.) with a sensitivity limit of <50 ng/mL.

Before antioxidant capacity analyses, serum was deproteinated according to a published method using methanol:acetonitrile:acetone (1:1:1, by vol) added to samples in a ratio of 1:4 (vol:vol) (29). This method allows for detection of small-molecular-weight antioxidants (<6 kDa). The antioxidant capacity was measured using the oxygen radical absorbance capacity assay on a FLUOstar Optima plate reader (BMG Labtech) (30). The compound 2,2-azobis (2-amidino-propane) dihydrochloride was used as the peroxyl radical generator and Trolox, a water-soluble analog of vitamin E, served as the reference antioxidant standard. Antioxidant capacity is expressed as micromole Trolox equivalents.

### Assessment of SNP genotyping

A thorough review of the literature was conducted to identify SNPs associated with lipoprotein transport and carotenoid metabolism. Saliva samples were used to isolate DNA. The isolated DNA was used to genotype 13 SNPs (Table 1) as part of a larger array: Infinium multi-ethnic global-8 (Illumina Inc.).

### Statistical analysis

Sample size considerations were based on published data regarding the influence of tomato juice on serum lycopene (31). Expecting a mean change in serum lycopene of 0.1 µM with an SD of change of 0.025 µM and a significance level of 5%, there was 80% power to detect a significant change in lycopene with 15 participants. Statistical analysis was conducted using SAS software version 9.4 (SAS Institute).

Intent-to-treat analysis was conducted using generalized linear mixed models to examine the effect of the intervention (i.e., random assignment to 100% watermelon juice or placebo) on serum lycopene, lipids, and antioxidant capacity (32). The multivariable-adjusted models included prerandomization scores at both study periods (i.e., before and after the washout period). Given the homogeneity of the study population, the a priori statistical plan was not adjusted for the aforementioned potential influencers of lycopene bioavailability (age, race, sex, or BMI). Because participants were of the same biological sex and race, additional analyses were conducted with age and BMI as covariates in order to confirm the a priori statistical plan. Analysis of the genetic data included the Kruskal–Wallis test or the Wilcoxon test depending upon the genotypic frequencies present in this sample population. Results reported herein are reflective of mean ± SD values. Owing to the exploratory nature of these analyses, exact *P* values are presented along with associations and estimates that have *P* values <0.05.

## Results

**Table 2** presents demographic data as well as the distribution of outcome measures at baseline. It should be noted that 17 participants completed the study, yet only 16 consented to providing a saliva sample for genotyping. Thus, statistical analysis was conducted with data from 16 participants. Results from the mixed models revealed that, as opposed to the group randomly assigned to the placebo, those randomly assigned to watermelon juice had significantly higher circulating serum lycopene (β coefficient: 0.5475; 95% CI: 0.204, 0.891; *P* = 0.0028). This finding met the significance threshold of 0.05 even after accounting for multiple testing. In addition, the *P* value obtained from the permutation test confirms the statistical significance of the findings. The related Cohen's *d* effect size was computed in order to gain further insight into the clinical significance. Serum lycopene exhibited a significant treatment effect (0.85) with striking interindividual differences in lycopene response (**Figure 1**). Consumption of 100% watermelon juice did not result in significant changes in serum lipids or antioxidant capacity (**Table 3**).

**TABLE 2:** Characteristics of participants at baseline^1^

Characteristics	Values
Demographics	
Age, y	60 ± 4.1
BMI, kg/m^2^	25.08 ± 3.6
Ethnicity, %	
Hispanic or Latino	0
Not Hispanic or Latino	100
Race, %	
White	100
Education level, *n*	
High school	2
College/postgraduate	14
Serum measures	
Lycopene, µM	1.37 ± 0.8
oxLDL-C, µg/dL	75.24 ± 10.4
Total cholesterol, mg/dL	218.78 ± 35.7
Triglycerides, mg/dL	98.56 ± 37.6
LDL-C, mg/dL	126.34 ± 34.4
HDL-C, mg/dL	73.59 ± 11.8
T-ORAC, µM TE	749.51 ± 231.3
H-ORAC, µM TE	251.07 ± 94.1
L-ORAC, µM TE	498.44 ± 153.4

1 *n* = 16. Values are means ± SDs unless otherwise indicated. HDL-C, HDL cholesterol; H-ORAC, hydrophilic oxygen radical absorbance capacity; LDL-C, LDL cholesterol; L-ORAC, lipophilic oxygen radical absorbance capacity; oxLDL-C, oxidized LDL cholesterol; TE, Trolox equivalents; T-ORAC, total oxygen radical absorbance capacity.

**TABLE 3:** Changes in serum lipids and antioxidant capacity after 4-wk consumption of 100% watermelon juice^1^

Serum measures	Pre–watermelon juice consumption	Post–watermelon juice consumption
oxLDL-C, µg/dL	72.41 ± 9.50	72.44 ± 8.88
Total cholesterol, mg/dL	205.07 ± 26.05	214.81 ± 32.58
Triglycerides, mg/dL	81.00 ± 28.46	97.50 ± 43.54
LDL-C, mg/dL	115.47 ± 27.64	124.38 ± 32.89
HDL-C, mg/dL	73.40 ± 12.60	70.94 ± 11.51
T-ORAC, µM TE	769.25 ± 251.77	869.66 ± 209.04
H-ORAC, µM TE	273.39 ± 99.06	328.99 ± 94.04
L-ORAC, µM TE	495.86 ± 204.62	540.67 ± 214.89

^1^ *n* = 16. Values are means ± SDs. Intent-to-treat analysis was conducted using generalized linear mixed models albeit no significant differences in serum lipids or antioxidant capacity were observed. HDL-C, HDL cholesterol; H-ORAC, hydrophilic oxygen radical absorbance capacity; LDL-C, LDL cholesterol; L-ORAC, lipophilic oxygen radical absorbance capacity; oxLDL-C, oxidized LDL cholesterol; TE, Trolox equivalents; T-ORAC, total oxygen radical absorbance capacity.

Among genetic variants previously shown to influence lycopene metabolism and bioavailability, 13 SNPs were associated with lycopene response (**Table 4**); however, only rs6564851 in the β-carotene 15,15’-oxygenase-1 (*BCO1*) gene was identified as significantly associated with changes in lycopene (β ± SE: 13.4 ± 1.6, *P* = 1.4 × 10^−06^).

**TABLE 4:** Distribution of genetic variants related to carotenoid metabolism and transport^1^

Gene	SNP	Chromosome (loc)	Genotypes (*n*)		
*LPL*	rs7841189*	8 (19845376)	TT (1)	TC (4)	CC (10)
*ELOVL2*	rs9468304*	6 (11042165)	GG (7)	AG (6)	AA (2)
*SOD2*	rs9365046	6 (159756737)	GG (13)	AG (2)	—
*ELOVL2*	rs911196*	6 (10990751)	AA (7)	AC (6)	CC (2)
*BCO1*	rs6564851*	16 (81264597)	GG (4)	TG (9)	TT (2)
*CD36*	rs4112274	7 (80167254)	CC (11)	TC (4)	—
*ABCG2*	rs1871744	4 (89039629)	TT (13)	TC (2)	—
*LPL*	rs17482753*	4 (19832646)	GG (11)	TG (3)	TT (1)
*MTTP*	rs17029173	4 (100509321)	TG (3)	TT (12)	—
*SCARB1*	rs1672879	12 (125281102)	CC (13)	AC (2)	—
*SCARB1*	rs11057841	12 (125316743)	CC (11)	TC (4)	—
*APOB*	rs1042031	2 (21225753)	GG (11)	AG (4)	—
*ABCB1*	rs10248420	7 (87164986)	AA (8)	AG (7)	—

1 SNP = single nucleotide polymorphism;*LPL = lipoprotein lipase; ELOVL2 = ELOVL fatty acid elongase 2; SOD2 = superoxide dismutase 2; BCO1 = beta-carotene oxygenase 1; CD36 = CD36 molecule; ABCG2 = ATP binding cassette subfamily G member 2; MTTP = microsomal triglyceride transfer protein; SCARB1 = scavenger receptor class B member 1; APOB = apolipoprotein B; ABCB1 = ATP binding cassette subfamily B member 1*.

* SNP analyzed by combining 2 of the 3 genotypes owing to the low representation of 1 genotype in this sample population.

Of interest, this significant change was observed in the placebo arm of the study with a mean change of 14.78 ± 1.95 µM serum lycopene among individuals with the TT genotype compared with a mean change of 1.42 ± 2.11 µM serum lycopene among the combined GG + TG genotypes. Two additional SNPs were found to be marginally associated with changes in lycopene in the placebo arm. These were rs9365046 in the superoxide dismutase 2 (*SOD2*) gene (β coefficient: 7.51, *P* = 0.049) and rs1871744 in the ATP binding cassette subfamily G member 2 (*ABCG2*) gene (β coefficient: 7.51, *P* = 0.049).

## Discussion

In light of evidence suggesting that lycopene may favorably affect blood lipids and redox balance, this study examined the effect of 100% watermelon juice supplementation on serum lycopene, lipids, and antioxidant capacity along with conducting an exploratory analysis of select SNPs related to lycopene metabolism and bioavailability. Despite significant increases in serum lycopene postsupplementation, a treatment effect on blood lipids or antioxidant capacity was not observed. Results are supported by previous findings in which a similar dose of lycopene provided by watermelon or tomato juice did not result in improvements in antioxidant capacity or cholesterol levels in middle-aged adults (33). In contrast, 2 recent systematic reviews reported varying degrees of improvement in lipid profiles after consumption of 100% fruit juice or supplementation with lycopene (34, 35). It is plausible that the discrepancy in results may be attributed to differing bioactive compounds as well as the dose and duration of the intervention. For example, in a recent meta-analysis of 21 intervention studies investigating lycopene and cardiovascular outcomes, a high degree of variability in treatment effectiveness was observed as a result of varying doses of lycopene as well as duration of the interventions ranging from 6 h to 6 mo (14).

Significant increases in circulating lycopene after consumption of watermelon juice are supported by previous intervention studies (18, 36, 37). Despite the significant overall treatment effect, wide interindividual differences in circulating lycopene were observed. In the present study, efforts were instituted to ensure that this variability was not attributable to intervention compliance or background diet. For example, adherence to beverage consumption across study arms was 92%, thus exceeding the minimal adherence threshold of 70%; furthermore, participant adherence to the low-lycopene diet was monitored throughout the study with an average lycopene intake across the study of 1.78 ± 3.24 mg/d. As a reference point, this is far below the average daily intake of lycopene in the United States (6.15 mg/d), Australia (3.8 mg/d), France (4.8 mg/d), or the Netherlands (4.9 mg/d) (38). To further elucidate the variability in serum lycopene in response to the intervention, this study sought to investigate genetic influences on lycopene metabolism and bioavailability. Indeed, a candidate SNP (rs6564851) in the *BCO1* gene was identified as significantly associated with changes in lycopene. These results are supported by findings from the InCHIANTI Study and the Women's Health and Aging Study in which the G allele of rs6564851 was significantly associated with lower circulating lycopene (*P* = 0.003) (19). Similarly, the G allele in this same SNP was significantly associated with lower circulating lycopene in a study investigating a tomato–soy juice intervention among men with prostate cancer (*P* = 0.046) (17). In the present study, the significant change in lycopene associated with this genetic variant was observed in the placebo arm only. Because lycopene has been shown to accumulate differentially in tissues, these findings may be partially explained by complex metabolic processes involved in fast-turnover tissues of lycopene (i.e., liver) compared with slow-turnover tissues (i.e., adipose tissue) (39–41). Further investigation is warranted to fully elucidate the influence of turnover tissues in releasing lycopene during periods of restricted or low-lycopene diets. Although the 2-wk washout period between arms was based on previous studies (42), a washout period of 2 wk may be insufficient if lycopene is indeed sequestered in adipose for release during periods of low lycopene intake.

Our study provides insightful results, yet the homogeneity of participants may limit the generalizability of these findings. For example, SNPs in other genes not presenting as significant in this analysis have been shown to predict serum lycopene differently based on ethnicity (12). Although the study was powered for the a priori analysis of circulating lycopene, the small sample size is certainly a limitation for genetic analysis. This study was strengthened by use of a cross-sectional design as well as by the use of gold-standard methodology for biochemical assays and SNP genotyping.

In conclusion, results from this study support the role of 100% watermelon juice in improving circulating lycopene. The daily provision of 100% watermelon juice bolstered lycopene intake above the reported average daily intake in various studied populations (38). This is a point of interest considering the previously acknowledged cardioprotective effects of lycopene coupled with the fact that lycopene intake has been reported to reduce one's risk of developing metabolic syndrome (35). Lastly, individuals’ responses to lycopene vary and it has been shown that a weak response to dietary lycopene based on the presence of other SNPs in the *BCO1* gene (rs12934922 and rs7501331) may be partially overcome by increasing the dose of lycopene provided in watermelon juice (18). Taken collectively, this indicates that future investigations with varying doses of lycopene-rich foods and SNP genotyping are warranted to establish individualized nutrition recommendations for conferring cardiovascular protection.

## Notes

Supported by American Heart Association Mentored Clinical and Population Research Award #16MCPRP27260233 (to KMC-W and ACE), National Institute on Aging Translational Nutrition and Aging Research Academic Career Leadership Award K07AG043588 (to JLL), and National Institute of Diabetes and Digestive and Kidney Diseases Center Core Grant DK056350 (to VSV).

Author disclosures: The authors report no conflicts of interest.
